# Use of a Mobile App to Facilitate Blood Glucose Monitoring in Adolescents With Type 1 Diabetes: Single-Subject Nonrandomized Clinical Trial

**DOI:** 10.2196/diabetes.8357

**Published:** 2018-02-07

**Authors:** Edward J Bellfield, Lisa K Sharp, Yinglin Xia, Ben S Gerber

**Affiliations:** 1 Department of Pediatrics University of Illinois at Chicago Chicago, IL United States; 2 Institute for Health Research and Policy University of Illinois at Chicago Chicago, IL United States; 3 Department of Pharmacy Systems, Outcomes and Policy University of Illinois at Chicago Chicago, IL United States; 4 Department of Medicine University of Illinois at Chicago Chicago, IL United States

**Keywords:** type 1 diabetes, adolescence, mobile health, mHealth, mobile phone

## Abstract

**Background:**

Cloud-based glucose monitoring programs allow users with diabetes to wirelessly synchronize their glucometers to their mobile phones. They also provide visualization and remote access of their data through its mobile app. There have been very few studies evaluating their effectiveness in managing diabetes among adolescents with type 1 diabetes (T1D).

**Objective:**

The purpose of this study was to assess the feasibility of using a mobile app to improve daily average blood glucose (BG) levels and increase BG monitoring frequency.

**Methods:**

We used an ABA single-subject prospective study design. We recruited five participants aged 13 to 17 years with uncontrolled T1D, glycated hemoglobin A1c 9.0%-10.7%, self-monitoring behavior of ≤5 checks/day, and on multiple daily insulin injections. The study consisted of 4-week intervals of three phases: (1) phase A: usual glucose monitoring log (fax); (2) phase B: mobile app; and (3) phase A': second phase A. A certified diabetes educator and endocrinologist reviewed logs and provided recommendations weekly. Data were analyzed using a quasi-Poisson model to adjust for overdispersion among individual participants, and a generalized estimating equation model for overall intervention effect in aggregate.

**Results:**

For mean daily BG (mg/dL) levels, participant 1 had decreased values on the mobile app (298 to 281, *P*=.03) and maintained in phase A'. Participant 4 had an increase in mean daily BG in phase A' (175 to 185, *P*=.01), whereas participant 5 had a decrease in mean daily BG in phase A' (314 to 211, *P*=.04). For daily monitoring (checks/day), participant 3 increased in phase B (4.6 to 8.3, *P*=.01) and maintained in phase A'. Participant 5 also had increased daily monitoring at each phase (2.1 to 2.4, *P*=.01; 2.4 to 3.4, *P*=.02). For the five participants combined, the overall mean BG and BG checks per day in phase A were mean 254.8 (SD 99.2) and mean 3.6 (SD 2.0), respectively, mean 223.1 (SD 95.7) and mean 4.5 (SD 3.0) in phase B, and mean 197.5 (SD 81.3) and mean 3.7 (SD 2.1) in phase A'. Compared to phase A, mean glucose levels declined during phase B and remained lower during phase A' (*P*=.002). There was no overall change in BG checks by phase (*P*=.25). However, mean BG levels negatively correlated with daily BG checks (r=–.47, *P*<.001). Although all participants had positive opinions about the app, its utilization was highly variable.

**Conclusions:**

We demonstrated modest feasibility of adolescents with uncontrolled T1D utilizing a glucose monitoring mobile app. Further study is needed to better determine its effects on BG level and monitoring frequency. Psychosocial factors and motivational barriers likely influence adoption and continuous use of technology for diabetes management.

## Introduction

In adolescents with type 1 diabetes (T1D), barriers to appropriate self-management abound [1]. It is a period of transition from childhood to adulthood, which is associated with multiple psychosocial stressors. As a result, adolescents with T1D have the worst glycemic control of all age groups, averaging a glycated hemoglobin A_1c_ (HbA_1c_) of 9% [2]. This finding is extremely worrisome because this increases the risk of long-term complications [3].

Self-monitoring is a critical component of T1D care. Multiple cohort studies show an association between frequency of glucose checks and better glycemic control when adjusted for age [4-6]. Although not a causal relationship, given that effective diabetes management includes insulin dosing, frequent blood glucose (BG) checking appears to be related to global self-care behavior, signifying that those who monitor BG more frequently are more likely to engage in good self-care [4]. Thus, there is compelling evidence to support self-monitoring with frequency depending on individual patient needs and goals [7]. Additionally, glucose monitoring is more likely to decline with age among adolescents with specific characteristics, such as residing in low socioeconomic households, having lower self-esteem, experiencing more stressful life events in the past year, and having a poorer quality relationship with parents or receiving less parental support [4].

Pediatric endocrinologists utilize manual logs of BG, insulin doses, and carbohydrate intake to determine insulin adjustments. However, if a patient uses multiple glucometers (eg, for use at home, school, daycare), in our clinical experience, it is unlikely that all the information will be logged to share with clinicians. Furthermore, based on our clinical experience, adolescents find that logging is an arduous task and thus will often not perform it of their own volition and less frequently as recommended by their physicians. Mobile phone apps can facilitate this process by automatically uploading BG, insulin dose, and carbohydrate intake data using wireless Bluetooth technology to a central Internet-based account accessible by authenticated providers. In fact, preliminary studies of Bluetooth-enabled self-monitoring devices in adults with type 2 diabetes and hypertension have shown promise of improved disease control [8].

Despite much publicity and marketing by app developers, there is very limited published data on the efficacy of mobile health apps in adolescents with T1D [9,10]. Additionally, rigorous research into clinical effectiveness of diabetes app designs in adolescents is lacking [11]. To date, there are at least two clinical studies that specifically evaluate mobile phone apps in adolescents with T1D. One pilot study showed an app with gamification incentives resulted in increased daily average frequency of BG measurements [9]; a second retrospective study of 81 adolescents showed that a glucometer mobile app increased monitoring frequency, particularly among those who synchronized their devices [12].

In this study, we prospectively evaluate a glucose monitoring system to determine feasibility, adoption, and impact, measured via monitoring frequency and BG levels, among adolescents with poorly controlled T1D.

## Methods

### Participants

Recruitment of low-income, minority patients with T1D occurred in an inner-city academic pediatric endocrinology clinic. Patients were screened before their appointment visit via chart review. Inclusion criteria were age 13 to 21 years, T1D diagnosed at least 1 year prior to study enrollment, on a multiple daily injection regimen, HbA_1c_ of 8% to 12% within the previous 6 months, and a no-show rate to clinic of less than 50% in the prior 12 months. After their diabetes clinic visits, patients were approached by research staff for further screening requirements: average daily glucose checks five times or less per day over the prior 2 weeks and verification using the patient’s glucometer(s). In addition, the patient and guardian were required to own compatible mobile phones with Internet access, a compatible glucometer, and report no prior use of the mobile app.

A total of five participants and guardians each received US $32 over the course of the study for their participation. Participant characteristics of the enrolled patients are included in Table 1. The University of Illinois at Chicago Institutional Review Board approved the protocol.

**Table 1:** Participant characteristics.

Participant	Sex	Age (years)	Race/Ethnicity	Years with T1D	Initial study HbA_1c_ (%)	Baseline mean daily BG^a^ checks
1	Female	17	African-American	2	9.0	2.4
2	Female	14	African-American	4	10.7	1.9
3	Female	15	Hispanic	1	9.1	4.0
4	Male	14	Hispanic	8	9.0	4.5
5	Male	13	African-American/White	2	9.4	5.0

^a^BG: blood glucose.

### Materials

Glooko (Glooko, Mountain View, CA, USA; the “mobile app”) is an online-based diabetes management system that incorporates automatic mobile phone reminders, allows for visualization of glucose trends and levels, and provides access of data by caretakers and clinicians [13]. The mobile app includes MeterSync Blue (“syncing device”), a Bluetooth-enabled attachment for patients to upload data from their glucometers to their mobile phone and online account. The syncing device is compatible with a majority of Food and Drug Administration (FDA)-cleared glucometers and with mobile phones utilizing iOS (Apple, Cupertino, CA, USA) and Android (Google, Mountain View, CA, USA) operating systems [14]. In 2012, the FDA cleared the mobile app for marketing as a Class II product after a 510(k) premarket notification review [15]. It is also HIPAA compliant [16].

At the start of the intervention period, participants opened an account online and on their mobile phone, and were loaned a syncing device. Participants returned the device at the end of the study.

### Experimental Design

An ABA single-subject prospective study design was used. Each phase lasted 4 weeks. In phase A, participants performed usual BG monitoring and were instructed to notate BG levels, insulin dosing, carbohydrate intake, and relevant activity in the clinic’s standard logbook. They were instructed to fax their logs to the clinic. A certified diabetes educator (CDE) called them once a week to discuss the log data and make recommendations as clinically indicated in consultation with the pediatric endocrinologist. At the end of phase A, participants and their guardians returned to the clinic to open the mobile app accounts. They downloaded the app onto their mobile phones, were taught the features of the program, and received the syncing device with instructions on use.

During phase B, participants performed usual BG monitoring but were instructed to synchronize their meter nightly and enter insulin dosing, carbohydrate intake, and relevant physical activities into the app. Each week, the CDE called participants to discuss the electronic log data and make recommendations as clinically indicated in consultation with the pediatric endocrinologist. The CDE and endocrinologist accessed an online dashboard that provided individual BG levels and descriptive statistics for review. The dashboard included graphical representations of BG logs, which included BG levels, carbohydrate intake, insulin administration, and percentage of time spent within BG goal.

At the end of phase B, participants were instructed to stop using the mobile app and restart manual logging with continued weekly CDE calls. Mobile app accounts were not suspended, but we were able to determine if the syncing device was being used. We emphasized to participants that the second phase A could not begin until cessation of synchronizing activity. A semistructured phone survey was also conducted to obtain feedback about their experience using the mobile app.

### Statistical Analysis

Study data were collected and managed using REDCap electronic data capture tools (REDCap, Nashville, TN, USA) hosted at the University of Illinois at Chicago [17]. Mobile app data were downloaded from the app’s clinician dashboard. Descriptive statistics for continuous variables were expressed as mean and standard deviation; categorical variables were presented as frequency and proportion. The intervention effect was reported as estimated mean and *P* values. All statistical tests were two-sided. First, we analyzed each of the five participants case by case. For each case, the scatterplots of both outcomes (BG level and number of BG checks) over time for the entire study period were generated to visually evaluate the patterns in the data with a smoother filter. The outcomes were analyzed using an interrupted time-series regression. We used both a quasi-Poisson and generalized least squares model. The quasi-Poisson model accounted for overdispersion by allowing the variance to be proportional rather than equal to the mean, whereas the generalized least squares model accounted for autocorrected residuals. Quasi-Poisson model results are reported. Then, we performed an overall correlation analysis for BG level and number of daily BG checks including all five participants together. Finally, we conducted a generalized estimating equation (GEE) analysis of intervention effect and association between BG level and number of daily BG checks through the SAS GENMOD procedure. GEE provides more robust inference to account for large variability [18]. All statistical analyses were conducted by R Core Team (R Foundation for Statistical Computing, Vienna, Austria) [19] and SAS version 9.4 (SAS Institute Inc, Cary, NC, USA).

## Results

Nine patients fulfilled the inclusion criteria, but one patient declined participation due to lack of interest. Eight provided assent/consent, and five completed the study. One male patient believed the study was too intrusive and dropped out after 4 weeks. Two other male patients could not commit to regular contact with the CDE and each dropped out after 2 weeks.

**Figure 1:**
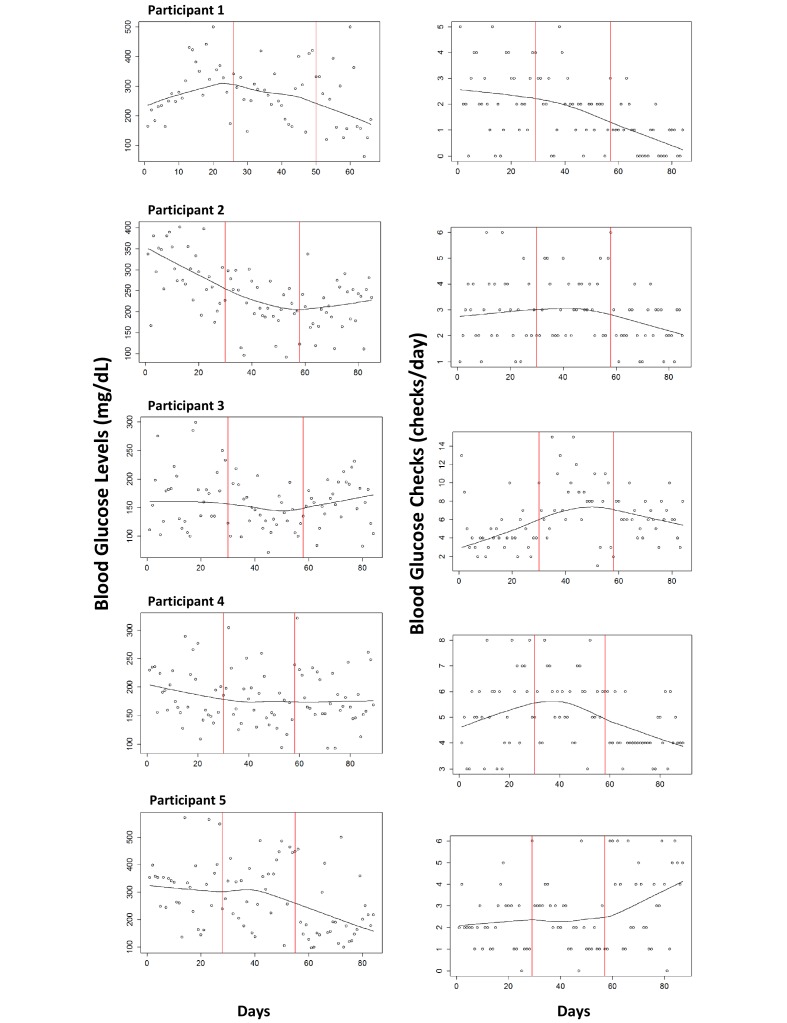
Mean daily blood glucose checks and levels over the course of the study period. The vertical lines denote the different phases.

Table 2 demonstrates the mean daily frequency of BG testing and mean daily BG levels. Figure 1 is a collection of time-series graphs showing both mean daily frequency of BG checks and BG levels for each participant.

Participant 1 had no significant changes in BG monitoring. However, BG decreased from phase A to B (298 to 281 mg/dL, *P*=.03) and was maintained in phase A'. Participant 2 had no significant changes in BG monitoring or mean BG levels. Participant 3 increased daily monitoring from phase A to B (4.6 to 8.3 checks/day, *P=*.01), and maintained in phase A'. However, there was no change in mean BG levels. Participant 4 had no significant changes in BG monitoring. However, mean daily BG levels increased from phase B to phase A' (175 to 185 mg/dL, *P*=.01). Participant 5 had increased daily monitoring from phase A to B (2.1 to 2.4 checks/day, *P*=.01) and again from phase B to phase A' (2.4 to 3.4 checks/day, *P*=.02). Furthermore, there was a decrease in mean daily BG levels from phase B to phase A' (314 to 211 mg/dL, *P=*.04). In aggregate, there was no significant difference in BG monitoring across phases (*P*=.25).

The frequency of hypoglycemia (BG level <70 mg/dL) paralleled the participant’s frequency of BG checks—the more checks, the more often hypoglycemia was discovered, as shown in Table 3. Data entry of insulin dosing, carbohydrate intake, and physical activity was inconsistent and limited, as shown in Table 3. Insulin adjustments were only made on two participants during phase A and phase A' predominantly because insulin and carbohydrate intake was more consistently noted on manual logs. The frequency of synchronization events in phase B are also shown in Table 3. There was maximum synchronizing in the first and second week, then it decreased thereafter.

For the five participants combined, the overall mean BG and BG checks per day in phase A were mean 254.8 (SD 99.2) and mean 3.6 (SD 2.0), respectively, mean 223.1 (SD 95.7) and mean 4.5 (SD 3.0) in phase B, and mean 197.5 (SD 81.3) and mean 3.7 (SD 2.1) in phase A'. Mean BG level was negatively correlated with daily BG checks (*r*=–.47, *P*<.001). GEE modeling confirmed the negative correlation between BG level and BG check frequency (*P*=.02); compared to the initial control period (A), the mean glucose values significantly decreased during the intervention phase (B; parameter estimate: –55.68, 95% CI –95.13 to –16.23) and maintained at reduced level (A'; parameter estimate –32.02, 95% CI –55.24 to –8.80, *P*=.002).

All five participants expressed a positive experience during interviews after phase A' period. Comments included: “I didn’t have to fax stuff, the app was easy to use,” “it’s easier than writing the numbers and the amounts of insulin by hand,” “it only takes about 3 minutes,” and “I really like it and would recommend it to anyone.” One participant indicated an increase in motivation to self-check glucose with automatic recording of levels, when previously she checked only at mealtimes. There were some technical issues, which affected the timeliness of the synchronizing process. Comments included: “I disliked that syncing takes too long,” “sometimes the app messes up and I have to turn it off and on again,” and “hard to Bluetooth it over because you have to hold it a certain way.” One participant suggested having a pop-up reminder for input of insulin/carbohydrate data when synchronizing. The pediatric endocrinologist and CDE both noted that the mobile app clinician dashboard was convenient to use and appropriately summarized the relevant data.

**Table 2:** Mean daily blood glucose levels and daily blood glucose checks.

Measure			Participant				
			1	2	3	4	5
**Blood glucose level (mg/dL), mean (SD)**							
	Phase A		298.2 (91.7)	295.9 (68.0)	175.1 (55.8)	190.1 (45.9)	325.4 (115.3)
	Phase B		281.4 (83.0)	215.2 (58.6)	141.4 (36.2)	174.5 (47.4)	314.5 (110.7)
	Phase A'		235.9 (119.6)	210.4 (57.5)	160.7 (38.5)	184.9 (50.0)	210.5 (112.7)
	**Difference, OR (95% CI)**						
		Phase B-A	0.71 (0.52-0.97)^a^	0.99 (0.77-1.26)	0.82 (0.61-1.10)	1.27 (0.99-1.62)	0.76 (0.52-1.11)
		Phase A'-B	1.13 (0.70-1.81)	1.02 (0.77-1.37)	1.23 (0.94-1.60)	1.46 (1.12-1.90)^a^	0.63 (0.41-0.98)^a^
**Blood glucose checks (checks/day), mean (SD)**							
	Phase A		2.7 (1.2)	3.0 (1.4)	4.6 (2.5)	5.3 (1.6)	2.2 (1.1)
	Phase B		2.3 (1.0)	3.2 (1.1)	8.3 (3.4)	5.6 (1.2)	2.5 (1.4)
	Phase A'		1.4 (0.7)	2.4 (1.1)	6.1 (2.0)	4.3 (0.9)	3.5 (1.9)
	**Difference, OR (95% CI)**						
		Phase B-A	1.34 (0.73-2.44)	0.97 (0.63-1.50)	2.42 (1.47-4.01)^a^	0.88 (0.69-1.14)	2.33 (1.33-4.11)^a^
		Phase A'-B	1.33 (0.77-2.30)	0.83 (0.45-1.53)	0.83 (0.54-1.26)	0.83 (0.66-1.03)	2.24 (1.17-4.30)^a^

^a^*P*≤.05.

**Table 3:** Frequency of detected hypoglycemia, mobile app data input, and faxing and synchronization events.

Measure		Participant				
		1	2	3	4	5
**Hypoglycemia detected, n**						
	Phase A	1	0	16	15	0
	Phase B	2	3	41	26	2
	Phase A'	1	4	22	10	0
**Mobile app data input**						
	Total BG^a^ checks, n	54	90	233	154	68
	Insulin notation, n (%)^b^	0 (0)	34 (38)	47 (20)	0 (0)	19 (28)
	Carbohydrates notation, n (%)^b^	2 (4)	71 (79)	58 (25)	3 (2)	29 (43)
	Manual input (ie, exercise activity, meal description, notation of pre/post meal), n (%)^b^	1 (2)	0 (0)	10 (4)	39 (25)	59 (87)
**Faxing and sync events**						
	Sent faxes (max 8), n	0	1	4	1	2
	Sync events in phase B, total (n per week)	27 (10/6/10/1)	5 (2/0/1/2)	7 (2/3/1/1)	7 (2/2/2/1)	22 (9/5/3/5)

^a^BG: blood glucose.

^b^Percentages were determined using the number of notations divided by total BG checks.

## Discussion

To our knowledge, this is the first ABA design to evaluate change in glucose monitoring frequency and BG levels from utilizing a mobile app in adolescents with T1D. A single-case (also known as “n-of-1”) study design was used because it can provide an efficient way to evaluate the effects of a behavioral intervention [20]. This study demonstrated modest feasibility of adoption of the mobile app. Overall, BG levels of the five participants declined from phase A to B, and remained lower during phase A'. Furthermore, lower BG levels were associated with more frequent BG checks, which mechanistically supports the rationale for increased monitoring. However, it still remains uncertain if improvement in mean BG levels are secondary to Glooko monitoring versus time and regression to the mean in this limited sample. Visualizations of the ABA graphs (Figure 1) describe outcomes that reflect individual monitoring behavior but cannot reveal other challenges influencing behavior and motivation, including psychosocial stressors and mood conditions. Further study is needed to better determine the app’s impact on BG levels and monitoring frequency.

Our study had mixed results with the usage of mobile app features. Although qualitative results suggest that participants preferred the mobile app to manual logging, there was variable use of app options and features. For example, participant 1 synchronized but rarely entered manual information. On the other hand, the rest of the participants preferably used either the insulin/carbohydrate function (manual numerical entry) or pre/post meal function (push button function). The effort required for manual data entry may inhibit complete logging [21]. The advantage of mobile apps is the elimination of certain tedious tasks (ie, logging). So for adoption of the technology, any steps that are substituted or added (ie, synchronizing, manual entry of non-BG data) has to be sufficient to promote motivation for use. It was time consuming and challenging for some to routinely synchronize the device, requiring close proximity between Glooko and the meter while the app remained open. The use of smart glucometers that do not require a syncing device and even integrating Bluetooth-enabled insulin pens to automatically notate dosage administration can potentially increase adoption of mobile health devices in diabetes care.

In our cohort, due to incomplete logging and lack of clinical indication, there was limited insulin dosing change recommendations in phase B. Only participant 3 had a BG target adjustment due to her multiple hypoglycemic readings.

All participants preferred using the mobile app to manual logging and faxing. In fact, although participants were supposed to fax a total of eight times during the study, most were unable to do so due to difficulty accessing a fax, especially during the summer when the school’s fax was not accessible. The most diligent participant faxed only 50% of the time recommended. This finding suggests that optimal use of Web-based apps can allow for more consistent review of data by clinicians.

This study demonstrates modest feasibility of adoption. Our cohort from populations of inner-city minority groups has the most to gain from effective technology as they typically experience worse outcomes.

Limitations of this study included the short length of the study (12 weeks). Furthermore, the study did not evaluate temporal effects (eg, school and other schedule changes) and confounders (eg, mood disorders, use of other diabetes apps management concurrently). Sampling bias, a limitation preventing generalizability, was also an intended feature of the recruitment process. The limited sample size also does not allow for definitive or even generalizable results. However, the results provide a realistic representation of actual short-term device use in a systematic evaluation.

Mobile health is not a panacea for chronic disease management because device use is tied to individual motivation to use it, extra effort involved in its use, etc [22]. There are certain patients who will not want to engage with the system, as was the case with four patients who refused to participate or dropped out of the study. Of those for whom there is high enthusiasm with novelty, technology does not necessarily translate to meaningful utilization or behavioral change. These technologies still do not overcome the underlying discomfort and inconvenience of BG monitoring. For mobile health devices to be clinically useful, facilitation of management has to overcome motivational barriers. In other words, significant psychosocial barriers (eg, significant home chaos, poor mental health, low motivation) will reduce impact, even if there is ample access to these devices. The patient has to be motivated sufficiently to improve his/her health if we are to expect them to use technology for such goals. In addition, to achieve benefit from increasing monitoring frequency, there must be subsequent motivated action, including better self-management. Patients must respond to feedback on hypoglycemia and hyperglycemia, adhere to lifestyle and treatment, and receive insulin adjustment under provider guidance.

The use of a mobile app by adolescents with T1D can be used in a low-income clinical setting, and provide important clinical information to caretakers and clinicians. There was observable variation in BG monitoring behavior, BG levels, and access of mobile app functions. The degree of behavior change is likely dependent on a host of psychosocial factors, and thus targeting the most appropriate patients who will benefit from this type of intervention may be key to maximize its effectiveness.

## Abbreviations

BG: blood glucose
CDE: certified diabetes educator
FDA: Food and Drug Administration
HbA1c: glycated hemoglobin A1c
T1D: type 1 diabetes
